# Dual-Action Regenerative Therapies: Regeneration and Antimicrobial Effects of Platelet- and Marrow-Derived Biologics

**DOI:** 10.3390/biomedicines13112832

**Published:** 2025-11-20

**Authors:** Claire Yuan, Samuel P. Ang, Jamal J. Hasoon, Reda Tolba, Qing Zhao Ruan, Christopher M. Lam, Giuliano Lo Bianco, Paul J. Christo, Christopher L. Robinson

**Affiliations:** 1 Christ’s College, Cambridge University, Cambridge CB2 3BU, UK; 2 Department of Anesthesiology, Perioperative and Pain Medicine, Brigham and Women’s Hospital, Harvard Medical School, Boston, MA 02115, USA; 3 Department of Anesthesia, Critical Care, and Pain Medicine, McGovern Medical School, UTHealth, Houston, TX 77030, USA; 4 Pain Management Department in the Anesthesiology Institute, Cleveland Clinic Abu Dhabi, Abu Dhabi P.O. Box 112412, United Arab Emirates; 5 Warren Alpert Medical School, Brown University, Providence, RI 02912, USA; 6 Department of Anesthesiology, Pain, and Perioperative Medicine, University of Kansas Medical Center, Kansas City, KS 66160, USA; clam2@kumc.edu; 7 Anesthesiology and Pain Department, Fondazione Istituto G. Giglio Cefalù, 90015 Palermo, Italy; giulianolobianco@gmail.com; 8 Division of Pain, Department of Anesthesiology and Critical Care Medicine, The Johns Hopkins University School of Medicine, Baltimore, MD 21287, USA; pchristo@jhmi.edu

**Keywords:** platelet-rich plasma, bone marrow aspirate concentrate, autologous protein solutions, regenerative medicine

## Abstract

This review explores the dual regenerative and antimicrobial properties of platelet- and marrow-derived biologics, including platelet-rich plasma (PRP), bone marrow aspirate concentrate (BMAC), autologous protein solutions, and plasma fractions. These biologics, widely used in regeneration and tissue repair, offer multiplex bioactivity through growth factors, cytokines, and cellular components that promote healing while reducing infection risk. PRP and BMAC demonstrate significant regenerative effects in musculoskeletal conditions, wound healing, and cartilage repair, with platelets and leukocytes contributing antimicrobial peptides and immune modulation for more indirect regenerative mechanisms. Preparation methods, patient factors, and lack of standardization impact clinical outcomes and efficacy. While promising for reducing reliance on chronic pain medications and improving function, these therapies face limitations including inconsistent preparation and utilization protocols, limited long-term safety data, and regulatory challenges. Here, we review the need for consensus-building, standardized procedures, and robust research to optimize clinical integration and realize the full potential of biologic regenerative therapies in pain medicine.

## 1. Introduction

Regenerative medicine aims to replenish cells and tissues to promote healing and diminish pain for a range of degenerative and ischemic diseases [1]. In the context of pain medicine, such biological therapies target underlying structural and biochemical causes of pain such as tissue degeneration, nerve injury, and inflammation [2]. Therefore, these biologics may also reduce reliance on chronic pain medications and reduce associated side-effects while improving long-term function and quality of life [3]. While many regenerative therapies have gained prominence, including stem cell therapy, gene therapy, and tissue engineering, this review will focus on protein-based therapies. As proteins and small molecules play a crucial role in healing cascades, these biologics have great potential for both direct and indirect promotion of wound healing [1].

Much of regenerative medicine in the field of pain management has focused on products such as platelet-rich plasma (PRP), bone marrow aspirate concentrate (BMAC), and protein concentrates. With the option for autologous origins, these interventions pose a lower risk of immune rejection and disease transmission. Further, the multiplex bioactivity offered by the mixture of growth factors, cytokines, and more are advantageous for healing [4]. These interventions are also comparably less invasive than surgery, offering an attractive alternative for patients [4]. Notably, blood- and marrow-derived products contain platelets, mesenchymal stromal cells, cytokines, and other proteins and biomolecules that provide dual benefits of pain relief via regeneration and anti-bacterial properties, thereby not only reducing patient discomfort but also warding off infection while promoting tissue, bone, and cartilage regeneration.

## 2. Biological Basis of Regenerative Protein Products

Wound healing involves a series of complex and well-orchestrated events, proceeding through four distinct phases: hemostasis, inflammation, proliferation, and remodeling [5]. Through each of these phases, numerous protein products and signaling molecules play crucial roles in enabling and promoting recovery [5].

Immediately following tissue injury, blood vessel walls constrict, and the coagulation cascade is initiated to limit blood loss. Platelets, essential for hemostasis, degranulate and release their α-granules, which contain growth factors (GF) including platelet-derived growth factor (PDGF), epidermal GF (EGF), insulin-like GF-1, platelet factor-IV, and transforming growth factor-β (TGF-β) (Table 1) [5]. These proteins then attract and activate macrophages, endothelial cells, and fibroblasts to begin the wound healing cascade. The clot itself is composed of fibrin, fibronectin, vitronectin, thrombospondin, and von Willebrand factor [5].

Inflammation is split into two stages. The early inflammatory phase lasts 1–2 days and begins with the activation of the complement system, while the late inflammatory phase takes place during days 2–3 and is characterized by blood monocytes attracted to the wound site undergoing a phenotypic adjustment to become tissue macrophages [5]. During early inflammation, polymorphonuclear leukocytes prevent infection [5]. Attracted to the wound by TGF-β and other factors, polymorphonuclear leukocytes undergo margination and diapedesis and kill bacteria and other foreign particles through phagocytosis [5]. Macrophages play a crucial role during late inflammation, regulating cell repair by both functioning as phagocytic cells and producing the GFs responsible for smooth muscle and endothelial cell proliferation. They also regulate extracellular matrix (ECM) production by fibroblasts [5].

Approximately three days after injury, the proliferation phase begins, lasting for 2–4 weeks. During proliferation, fibroblasts are attracted to the wound by PDGF and TGF-β, among other factors [5]. Fibroblasts then proliferate, generating fibronectin, hyaluronan, proteoglycans, and collagen, matrix proteins that help produce the new ECM [5]. The proliferation phase is characterized by granulation tissue formation, extracellular matrix deposition, and migration of fibroblasts [5]. During the synthesis of granulation tissue, the basement membrane of the parent vessel is degraded to form a capillary sprout. Endothelial cells migrate to the wound, proliferate, and mature into capillary tubes [5]. Proliferation concludes with epithelialization, regulated by GFs including epidermal growth factor and basic fibroblast growth factor [5]. In the final phase of wound healing, remodeling and scar maturation over several weeks involves the continuous formation and degradation of collagen by metalloproteinases produced by fibroblasts, macrophages, and neutrophils in the wound. With scar maturation, fibronectin and hyaluronan are broken down and collagen bundles are thickened [5].

## 3. Platelet-Rich Plasma

PRP is a concentrate consisting of platelets, GF, cytokines, and other bioactive molecules. Derived from megakaryocyte fragments, platelets are small anucleate cells that play an important role in blood clotting during tissue and vascular injury [19]. Platelet cells contain dense, α, and lysosomal granules. The α granules feature GF, cytokines, cell adhesion molecules, proteins, and chemokines that are crucial for PRP function [20]. As an autologous blood product derived from the patient, PRP is designed to minimize allergy and rejection [19]. Though there remains little standardization in PRP preparation, studies have employed a variety of methods with varying quality and effectiveness [21].

### 3.1. Preparation and Activation

Studies have indicated the importance of evaluating individual patient and material conditions prior to the preparation of PRP [21]. Patients over the age of 60 may face reduced platelet availability for collecting due to thrombocytopenia [22]. Data on gender differences has largely been inconclusive [23,24,25]. Even choice of blood draw site and needle type may influence platelet recovery and viability [26].

No standardized method of PRP isolation exists, with variation dependent on clinical needs, desired PRP concentration and leukocyte content, cost, and other logistics [19,21]. The quality and efficacy of isolated PRP can be gauged through assessment of recovery rate (RR) and increase factor, though these do not always correlate with GF concentrations, which are critical for proper clinical outcomes [19,21]. Isolation techniques can be categorized generally as centrifugation-based, standard cell separators, and autologous selective filtration (apheresis) [27].

In centrifugation-based isolation, the blood separates by component density, with RBCs occupying the base layer, buffy coat platelets and leukocytes in the middle, and platelet-poor plasma (PPP) on top. Commercial kits provide closed systems designed for safety and ease of use but recover a lower fraction of platelets (40–60%), vary widely in cost, and may result in high RBC or leukocyte contamination [28]. In single-spin centrifugation, low *g*-forces (44–240× *g*) are used to maximize platelet recovery and GF concentrations while limiting cell damage. These protocols tend to yield higher leukocyte contamination, which may result in inferior bone regeneration effects in PRP [21,29]. Double-spin centrifugation is more commonly employed than single-spin centrifugation and features a low *g*-force separating spin to reduce RBC levels followed by a high *g*-force condensation spin to form a concentrated platelet pellet [21,30]. The double-spin centrifugation technique is preferred for enhanced control over PRP composition and high platelet recovery rates of up to 99% [31]. Still, published protocols show wide inconsistencies in handling differences, hematocrit, tube geometry, volume of whole blood, and plasma portion transferred between first and second spin, limiting comparability and standardization (Table 2) [21]. In standard cell separators, the patient’s blood is drawn and anticoagulated before undergoing continuous or discontinuous centrifugation. As a software-controlled procedure, standard cell separators present a precise and easy use method for generating large volumes of PRP [21].

Apheresis also offers a potential technique for generating high volumes of PRP. Also, a software-guided system, autologous selective filtration isolates blood components based on size and weight through continuous flow [21]. However, apheresis may cause platelet damage and activation as a result of contact with the filter membrane and shear stress [32]. Platelet activation can impact GF release and limit PRP product quality [33].

Following isolation, PRP can be used directly or undergo activation to stimulate greater GF release, though there remains limited consensus on whether pre-application activation is ideal [19,21]. Activation can be chemical or physical. In chemical activation, calcium chloride, thrombin, thrombin receptor agonist peptide, collagen, ADP, and chemically induced lysis can yield varying amounts of GF release, degrees of clot formation and inflammation, and other factors [34,35,36]. Physical activation techniques that stimulate platelets without chemical intervention, such as freeze-thawing and sonication, seek to avoid immune responses or toxic byproducts [37,38].

**Table 2:** Examples of differences across PRP preparation and activation protocols.

Spin Protocol	Blood Volume	Tube/Separator Type	Anticoagulant
Double: (1) 100× *g*, 15 min; (2) 1600× *g*, 20 min [31]	10 mL	10 mL conical tube	ACD-A
Single: 100× *g*, 10 min Double: (1) 100× *g*, 10 min; (2) 400× *g*, 10 min [39]	15 mL	Conical tubes	ACD-A
Single: 3000 rpm, 15 min Double: (1) 15,000 rpm, 6 min; (2) 2500 rpm, 15 min [40]	15 mL	Standard tubes	Sodium citrate 3.2%
Double: (1) 900× *g*, 5 min; (2) 1500× *g*; 10 min [41]	18 mL	15 mL conical tube	Citrate phosphate dextrose

### 3.2. Indications

PRP has indications in orthopedics, musculoskeletal pain conditions, dermatology, chronic wound healing, maxillofacial surgery, and urology, among others (Table 3) [19]. The evidence is strongest for positive outcomes for the use of PRP in pain and orthopedic conditions, including lateral epicondylitis, patellar tendinopathy, osteoarthritis (particularly of the knee), and other tendinopathies by boosting chondrocyte activity, ECM synthesis, and cartilage repair, as well as reducing inflammation [42,43].

Further, studies have reported PRP to be beneficial in chronic wound healing by promoting angiogenesis, granulation, and re-epithelialization [19]. PRP growth factors have also been shown to be beneficial in promoting bone formation and regeneration in various dental, maxillofacial, and spinal surgeries, as well as stimulating hair follicles for dermatological applications [19,51]. PRP studies have indicated low rates of serious adverse events, featuring complication rates between 1–3% [44,45]. Indeed, the autologous nature of PRP is designed to reduce the risks of disease transmission, immune reactions, and rejection compared to allogenic interventions [19,21].

### 3.3. Antibacterial and Antifungal Properties

Due to the high population of platelets and GFs, PRP demonstrates antimicrobial properties (Table 4). Activated platelets release antimicrobial peptides of kinocidins from α-granules, such as CXCL4, CXCL7, CXCL5, that can act against bacterial and fungal infections [52]. Platelets may also generate reactive oxygen species, resulting in cellular cytotoxicity through antibody-dependent mechanisms [53,54]. PRP has achieved 26–46% reduction in *S. aureus* activity in in vitro isolates from surgical site infections and in equine biofilms, as well as demonstrating log-reduction action against *E. coli*, *P. aeruginosa*, and *K. pneumoniae* [52,55,56]. Assessment of the antifungal activity of PRP has yielded more mixed results. In studies on oral pathogens, PRP has been shown to inhibit *C. albicans*, though it had limited results against other fungal pathogens [8,57]. However, autologous platelet concentrates have shown potential for use as effective antifungal drug carriers [58]. Platelet activation and concentration play an important role in modulating antibacterial and antifungal activity.

Leukocytes in PRP, particularly neutrophils and monocytes, also carry granules with defensins, lysozymes, cathelicidins, and others that possess antimicrobial activity. The cytokines produced by leukocytes can also serve in indirect regeneration, modulating inflammation and participating in further immune modulation [52]. For PRP injections, infection rates can be as low as <1%, making the biologic comparable to corticosteroid controls [45]. Still, the assessment of antibacterial and antifungal properties in PRP has largely involved preclinical and bench data, with limited clinical trial data and strength [52]. Further, it is important to note that low infection rates may simply reflect procedural safety rather than proven antimicrobial and antifungal activity [64].

### 3.4. Limitations

Despite its promise, significant limitations remain for the widespread use of PRP. Clinical outcomes remain inconsistent, and short follow-up times and small sample sizes limit understanding of the long-term safety and efficacy of PRP [19,21]. Further, heterogeneity in preparation, in addition to inadequate documentation of processes, provides a plethora of protocols with little agreement on which provide optimal results [65]. Similarly, many studies do not report the quantitative data on final PRP composition [65]. As such, this points to the continued need for standardization in the use of PRP in regenerative medicine.

Moreover, there remains limited regulatory oversight, commercial kit validation, and overall quality control related to PRP [66]. PRP has not received widespread regulatory FDA approval for clinical use. Though PRP is relatively lower cost compared to advanced biologics, its limited by little to no insurance coverage [66,67].

## 4. Bone Marrow Aspirate Concentrate

BMAC contains mesenchymal stem cells (MSCs), GFs, platelets, hematopoietic cells, and a range of cytokines. MSCs act through both indirect and direct regenerative mechanisms, such as rebuilding tissue by differentiating or inducing differentiation into a range of cell types like myocytes and fibroblasts [46]. BMAC has shown regenerative and immunomodulatory properties through paracrine signaling that has potential for cartilage and bone regeneration [47,48].

Through injection directly into a damaged area, BMAC can promote bone healing by differentiating into osteoblasts, reducing inflammation, and releasing GFs that further stimulate tissue repair [49]. In cartilage repair, BMAC provides elevated concentrations of GFs to stimulate the synthesis of chondrocyte ECM for direct regenerative effect [50]. Further, the elevation of GFs such as TGF-β1 and IGF-1 in BMAC may protect chondrocytes from catabolic activity [50]. Autologous or allogenic MSCs may be used in BMAC. Autologous MSCs are easier to harvest and reduce risk of rejection and disease transmission in use but require a two-stage preparation sequence [68]. While allogenic MSC preparations can be delivered in one stage, they demonstrate weaker evidence of long-term safety and efficacy [69,70].

### 4.1. Preparation and Delivery

Preparation of autologous BMAC can vary widely across aspiration sites, technique, and composition. Bone marrow is typically aspirated from the patient’s iliac crest, though other regions such as the proximal femur and tibia have been used clinically as well [71,72]. The composition of the BMAC is largely dependent on the subject’s biology, including age. Location and technique for harvesting can also impact MSC yield [73,74,75]. During aspiration, choice of needle gauge and syringe size, as well as the speed of plunger withdrawal, can influence the pressure applied to aspirate the MSC [76]. Further, the volume of marrow collected must be considered to ensure sufficient MSC population in the sample. Use of anticoagulant during aspiration to prevent clotting likewise ranges across protocols, including heparin and citrate dextrose [71,77].

The sample is then centrifuged to separate and concentrate cellular components to increase the viability and efficacy of MSCs and other bioactive factors [46]. As in PRP preparation, several centrifugation methods have been employed in the preparation of BMAC, including commercial kits, dual spin, and single spin, with similar strengths and limitations to use in PRP cell separation [46].

Injection protocols for the delivery of the prepared concentrate into the target site also persist, with studies showing varying preference for single vs. repeated administration and intra-articular vs. subchondral delivery [48]. While both single and repeated injection protocols reduce pain and improve function, repeated administration may offer improved patient outcomes over time [48]. However, repeated injections have the potential to cause immune responses and inflammation [78]. For therapeutic uses of BMAC in joints, the concentrate may be delivered directly into the joint through intra-articular injections or targeted to the osteochondral unit in subchondral injections [48]. In some studies, subchondral BMAC injections have been reported to show enhanced functional improvements and pain reduction over intra-articular injections [79,80].

### 4.2. Indications

BMAC has shown greatest indications for pain and musculoskeletal conditions (Table 3). In particular, multiple studies have demonstrated BMAC injections for knee osteoarthritis resulted in marked improvement in pain and patient outcomes at short- to mid-term follow-up [48,61,81]. Some studies have also suggested BMAC to promote positive outcomes in treatment of cartilage damage, though the evidence remains limited [50,71]. There is limited evidence for the efficacy of BMAC in certain indications, including spine disorders and degenerative disc disease [61]. BMAC, though autologous, is more invasive than PRP due to the need for intraosseous versus intravascular access.

### 4.3. Antibacterial and Antifungal Properties

BMAC contains bone marrow stromal cells (BMSCs), which have immunomodulatory effects and release antimicrobial peptides, acting through indirect regenerative mechanisms. BMSCs express hepcidin, which is upregulated in response to bacterial or other inflammatory agents [60]. BMSC-derived hepcidin displays antibacterial activity in vitro and reduced inflammation in vivo [60]. BMSCs also produce antimicrobial peptides such as LL-37, which has broad spectrum antibacterial activity and immunomodulatory roles [60,61]. LL-37 acts to inhibit *S. aureus*, *E. coli*, *P. aeruginosa*, among other bacterial infections [82]. Therefore, the degree of antibacterial activity present in BMAC depends on how rich the sample is in these cells and molecules. Though the evidence for antifungal activity in BMAC is weaker and poorly characterized, LL-37 was found to have some antifungal function against *C. albicans* in vitro [60].

Alongside specific extracts, BMAC also contains leukocytes, neutrophils, plasma proteins, and growth factors, which can contribute some degree of antibacterial and antifungal activity, as previously mentioned (Table 4). These bioactive factors are also involved in paracrine signaling to influence healing, angiogenesis, suppression of inflammation, cell survival, and modulation of immune cell behavior [83,84]. In BMAC, studies have indicated very low infection rates, though one study noted slightly elevated infection rates at 17.5% [85]. This study featured a cohort of 80 patients and a 7-year follow-up, marking a comparatively more long-term scope of assessment [86].

### 4.4. Limitations

Similarly to PRP, BMAC faces several limitations, particularly surrounding lack of standardization in harvesting and processing. Wide variability in site of harvesting, type of anesthesia, syringe size, anticoagulant employed, and centrifugation settings can greatly hinder the comparability of studies and reproduction of results [46,75,87].

Patients report pain during the aspiration procedure, which also holds risks of infection, hematoma, and bleeding at the aspiration site [88]. Though pain control during this procedure has not been standardized, the application of local anesthetics can reduce discomfort [89]. Studies have indicated no marked reduction in pain when local anesthetic agents were used, but incorporating intravenous sedation has reduced anxiety and pain perception in patients. There remains limited analysis of the relationship between use of anesthesia and MSC yield [46].

Further, many studies investigating BMAC are small, nonrandomized, and feature short follow-up times, further limiting conclusions [48,61]. Variations in MSC number and capacity—as well as other factors related to age and patient biology—also have limited broken down and collagen bundles are thickened [5].

## 5. Autologous Protein Solution and Plasma Fractions

### 5.1. Autologous Protein Solution

Autologous protein solution (APS) is a blood-derived protein biologic containing elevated levels of leukocytes, platelets, and plasma proteins [90]. Notably, APS contains greater proportions of platelet- and plasma-derived GF and cytokines compared to PRP [90]. Processed with commercial kits, the solution is produced by treating blood in an APS separator that sequesters white blood cells and platelets in a small amount of plasma before transferring to an APS concentrator for desiccation by filtration [90]. During processing, white blood cells are stimulated to produce more anti-inflammatory cytokines to yield the final enhanced concentration [91]. The solution can then be injected intra-articularly into the site of interest [92].

APS has shown to improve osteoarthritis in horses and dogs, enhancing joint mobility and lameness [90,93]. In humans, APS has revealed increased levels of interleukin-1 receptor 1 and tumor necrosis factor receptor 2 [94]. However, APS has not been studied as extensively as other hemoderivatives, so further research is necessary to fully evaluate clinical relevance and safety in humans [93,94].

Clinical use of APS has suggested that the solution increased M2 macrophages and decreased M1 pro-inflammatory macrophages, performing important functions in immune modulation [95]. Further, a pilot safety and efficacy study of APS in osteoarthritis showed it contained anti-inflammatory cytokines that can play some antimicrobial roles (Table 4) [63].

With limited study in humans, the clinical relevance of APS remains limited compared to biologics like PRP and BMAC. Nonetheless, it suggests promise in optimizing the wound environment and may be further studied as an indirect microbial agent with regenerative properties.

### 5.2. Platelet-Poor Plasma

PPP is often produced as a byproduct of the PRP isolation process [96]. While very low in platelet count, this remaining plasma still contains the typical soluble plasma proteins, including albumin, clotting factors like fibrinogen, immunoglobulins, and GF [96]. To prepare PPP, blood is drawn from the patient with the use of an anticoagulant [35]. As in aforementioned sections, the identity of the anticoagulant may vary across protocols. Following an initial centrifugation process to separate RBCs and buffy coat from the plasma, a second, stronger spin with higher *g*-forces or longer duration is employed to pellet remaining platelets [35].

In regenerative medicine, PPP has been explored for use in pain conditions and surgical settings. As PPP retains fibrinogen and clotting proteins, it can be converted upon activation into fibrin sealants or glues [97]. Despite its limitations in healing properties with the reduction in platelet count, PPP was shown to have some benefits in muscle injuries in athletes [98]. However, PPP is more commonly discussed in conjunction with PRP. For instance, adding PPP to PRP has an inhibitory effect on platelet aggregation during clot formation, which could be attributable to the high-speed centrifugation required to produce PPP [96].

Studies have reported mixed results on the antibacterial and antifungal properties of protein-poor plasma. PPP had no significant impact on the growth of bacteria compared to PRP, which inhibited bacterial proliferation [99]. There limited PPP antibacterial activity is attributed to the complement system, as PPP antibacterial activity declined significantly following heat inactivation [100].

PPP remains poorly studied compared to PRP and therefore faces significant limitations. Many applications of PPP remain small-scale, experimental, or observational, pointing to the need for robust randomized controlled trials [35,96,98]. Further, as the protocol for preparing PRP is not standardized, PPP also suffers from a lack of standardization in composition and isolation procedure [35].

Like APS, platelet-poor plasma may provide promise as an indirect immune support, particularly when used in clinical settings in conjunction with more robustly understood therapies like PRP. Despite limitations requiring standardization and further study, PPP may prove a safe and viable option adjacent to PRP.

### 5.3. Protein-Rich Plasma and Ultrafiltration-Derived Protein Concentrates

Rather than discarding or collecting PPP, ultrafiltration allows plasma water to be removed to obtain a higher concentration of plasma proteins and biomolecules [101]. Following blood draw, plasma and platelets are separated through centrifugation. Ultrafiltration is then performed using membrane filters of ultrafiltration units to remove plasma water and concentrate proteins, platelets, growth factors, and other biomolecules present in the sample [101]. A range of filtration membranes with varying size cutoffs exist and have been employed, from 3 kDa to 25 kDa, chosen to selectively retain biomolecules [101,102].

Ultrafiltration-derived protein concentrates are often used in conjunction with other platelet concentrates to generate protein-rich (PR-PRP) or protein-enriched filtered PRP (PEF-PRP) for clinical use. PR-PRP is particularly rich in plasma proteins like albumin, fibrinogen, immunoglobulins, and growth factors [103]. Following activation, PR-PRP is able to promote interactions with cells like macrophages, MSC, and fibroblasts, resulting in tissue reparation and healing [103]. In particular, PR-PRP employs a sustained release matrix that allows the prolonged release and presence of a highly concentrated sample of biomolecules and cells until the PR-PRP matrix is dissolved [103].

PEF-PRP contains increased concentrations of clot stabilizing and cell–matrix adhesion proteins that can create an environment to stimulate tissue regeneration [104]. One study reported positive results for the use of PEF-PRP to treat severe pressure ulcers [104]. In particular, researchers sought to use the enhanced concentration of platelets and plasma proteins in PEF-PRP to stimulate ECM regeneration without the need for plastic surgery [104].

As with other platelet concentrates, ultrafiltration-derived protein concentrates benefit from the antibacterial and antifungal properties exhibited by platelets and some plasma proteins and biomolecules. Additionally, plasma-derived growth factors such as IGF-1 and HGF possess anti-inflammatory effects [103].

Similarly to PPP, ultrafiltration allows for the production of PRP variants that can provide wound environment supportive functions to promote healing, which may prove invaluable in clinical applications.

## 6. Other Protein-Based Regenerative Therapies

Autologous conditioned serum (ACS) is a cell-free, blood-derived biologic that features increased concentrations of cytokines and GF [105,106]. To prepare the serum, blood is drawn and incubated to trigger the production of additional cytokines by white blood cells [106]. The sample is then centrifuged to collect the serum. Like APS, it is produced using commercial kits; however, ACS requires incubation [91]. ACS is typically applied via intra-articular injection, though it can also be included in wound dressings for topical application [107]. There remains variation in dosage, injection number and frequency, activation, and centrifugation protocols [108]. Indications for ACS include pain conditions, musculoskeletal injuries, degenerative diseases, and wound healing [105,109]. ACS has shown positive results in reducing pain, improving function, and slowing degradation in patients with knee osteoarthritis [109].

Additionally, functionalized biomaterials with antibacterial agents have demonstrated potential in regenerative medicine with broad use in pain medicine, orthopedics, dentistry, wound care, and implants. A wide range of potential materials have been explored, including metal–organic frameworks with antimicrobial surface coatings [110]. The metal-ion release scaffolds can act to enhance osteogenic differentiation, which may act to up-regulate genes, angiogenesis, and macrophage polarization for regenerative medicine and pain management [111]. The surface structure of the scaffolds, including nanotubes, nanospikes, and porous coatings can impact cell adhesion and blood clotting, again relevant in wound healing and regeneration [112]. For instance, copper-coated manganese dioxide nanoparticle action on osteogenesis revealed positive outcomes [113].

## 7. Conclusions

For clinical pain management in a wide spectrum of conditions and operations, regenerative medicine therapies such as PRP and BMAC offer significant future potential. Offering multi-pronged potential in pain reduction, tissue regeneration, and antimicrobial properties, these interventions signal the rise of a new era in pain medicine. However, significant practical limitations remain. In particular, the large variations across protocols for preparation, delivery, and characterization of biologics hinder systematic analysis and review, and the lack of research featuring large sample sizes and long follow-up times indicate a shortage of data surrounding long-term safety and efficacy. Further, clinicians require clearer guidelines on patient selection and dosages to optimize outcomes. Limited regulatory oversight also presents legal and ethical challenges.

For widespread adoption, notable efforts must be made towards consensus-building in the field to standardize procedures and quality metrics. Future directions must prioritize large-scale research and careful integration into clinical practice, considering not only long-term impacts but also regulatory oversight and financial barriers. With time, these therapies have the potential to usher in a paradigm shift towards more biological and holistic healing strategies for improved patient outcomes.

## Figures and Tables

**Table 1:** Key cellular and molecular components of wound healing with associated antibacterial/antifungal properties.

Type	Healing	Antibacterial/Antifungal
Cells		
Platelets	Thrombin formation	Release antimicrobial peptides [6] Generate reactive oxygen species [6] Direct antibacterial activity: *S. aureus*, *E. coli* [7,8] Limited antifungal properties
Leukocytes	Phagocytose bacteria and foreign particles	Release antimicrobial peptides [9] Generate reactive oxygen species [9]
Growth factors		
PDGF	Endothelial cell proliferation [5] Fibroblast proliferation and chemotaxis Smooth muscle cell proliferation Neutrophil chemotaxis	PDGF-derived peptides show antimicrobial activity [10]
VEGF	Stimulates granulation tissue angiogenesis [5] Stimulates blood vessel formation	Cell-mediated antimicrobial action: upregulation of autophagic, lysosomal pathways [11]
TGF-β	Fibroblast proliferation [5] Macrophage movement Smooth muscle cell proliferation	Modulate macrophage function against bacterial and fungal infections [12]
IGF-1	Fibroblast proliferation [5] Stimulates sulphated proteoglycan synthesis Stimulates collagen synthesis	Attenuate antimicrobial mechanisms [13] Little direct effect
HGF	Re-epithelialization [5] Neovascularization Granulation tissue formation	HGF-derived peptides have antimicrobial activity [10]
Plasma proteins		
Fibrinogen	Provisional ECM formation [14] Contribute to hemostasis Support re-epithelialization Support angiogenesis	Physical barrier against microbes [14] Modulate immune behavior through leukocyte binding
Albumin	GF transport [15] Maintain fluid balance	Antifungal action via fatty acids [16] Direct antibacterial activity [17]
Alpha-2-macroglobulin	Inhibit proteases [18] Bind and modulate GF, hormones, cytokines	Modulate inflammatory mediators [18]

**Table 3:** Summary of study design, outcomes, and potential adverse effects for clinical indications of PRP and BMAC.

Study Design	Outcomes	Safety/Adverse Events
PRP		
Systematic review and meta-analysis of RCTs in knee OA [44]	PRP reduced pain, improved function at 6 months compared to placebo and HA	High risk of bias across trials Limited evidence
Meta-analysis of RCTs [45]	PRP reduced pain, improved function at 12 months compared to placebo and HA PRP similar to HA at 6 months	PRP did not increase risk of adverse events
Preclinical in vivo study in animal models [29]	LP-PRP promoted bone repair better than LR-PRP	Human safety not studied
Observational study, cross-sectional [24]	Positive correlation between platelet count and GF levels in LP-PRP	Safety outcomes not applicable
BMAC		
Experimental, observational study [46]	Procedural variables (e.g., aspiration site, centrifugation speed and time) affect MSC yield	Limited as a process study
Systematic review of clinical studies in knee OA [47]	BMAC reduced pain, improved function in short term	BMAC safe in short term; limited data on long term safety Risk of bias in studies
Narrative review of knee OA [48]	BMAC relieves symptoms in short- and mid-term Possible cartilage and bone regeneration	BMAC appears safe but limited long-term data Inconsistent protocols
Animal model analysis [49]	BMAC enhances osteogenesis and angiogenesis	Human safety not assessed
Translational, clinical study review [50]	BMAC may promote cartilage repair	Limited clinical safety data

**Table 4:** Antimicrobial and antifungal properties in vitro and in vivo for PRP, BMAC, and APS.

In Vitro	In Vivo
PRP	
P-PRP inhibited *Enterococcus faecalis*, *Streptococcus oralis*, *Candida albicans* in agar tests; no action against *Pseudomonas aeruginosa* [8]	PRP and PRF seem to provide antimicrobial activity beneficial in clinical wound management [59] PRP reduced infections in animal models (e.g., equine, rabbit) [7,52]
BMAC	
Reduced bacterial proliferation; via hepcidin secretion and immunomodulation [60]	Clinical indications of indirect antimicrobial effect, regenerative support [61,62] Reduced bacterial load and inflammation in animal models (e.g., rat, rabbit) [60]
APS	
(Ex vivo) Indirect antimicrobial and antifungal activity in explants [11]	Positive joint tissue environment modulation; indirect antimicrobial effect [63]

## Data Availability

No new data were created or analyzed in this study. Data sharing is not applicable to this article.

## Abbreviations

The following abbreviations are used in this manuscript:

PRP	Platelet-rich plasma
BMAC	Bone marrow aspirate concentrate
GF	Growth factor
PDGF	Platelet-derived growth factor
EGF	Epidermal growth factor
TGF-β	Transforming growth factor-β
ECM	Extracellular matrix
RR	Recovery rate
PPP	Platelet-poor plasma
MSC	Mesenchymal stem cell
BMSC	Bone marrow stromal cell
APS	Autologous protein solution
PR-PRP	Protein-rich platelet-rich plasma
PEF-PRP	Protein-enriched filtered platelet-rich plasma
ACS	Autologous conditioned serum
